# Exploring network structure and central items of the Narcissistic Personality Inventory

**DOI:** 10.1002/mpr.1810

**Published:** 2019-12-05

**Authors:** Giovanni Briganti, Paul Linkowski

**Affiliations:** ^1^ Université Libre de Bruxelles Belgium

**Keywords:** biostatistics, methodology, personality disorder, psychometrics

## Abstract

**Objectives:**

The aim of this work is to explore the Narcissistic Personality Inventory (NPI) using network analysis in a dataset of 942 university students from the French‐speaking part of Belgium.

**Methods:**

We estimated an Ising Model for the forty items in the questionnaire and explored item interconnectedness with strength centrality. We provide in the supplementary materials the dataset used for the analyses as well as the full code to ensure the reproducibility of our results.

**Results:**

The NPI is presented as an overall positively connected network with items from *entitlement, authority* and *superiority* reporting the highest centrality estimates.

**Conclusions:**

Network analysis highlights new properties of items from the NPI. Future studies should endeavor to replicate our findings in other samples, both clinical and non‐clinical.

## 1 INTRODUCTION

Narcissism has been defined as the ability to maintain a positive self‐image despite various internal and external processes. Narcissistic subjects have a need for self‐enhancing experiences from their social environment (Pincus et al., 2009). Narcissism has been theorized to possess both normal and pathological aspects, which have been considered by some authors as two different personality constructs (Von Kanel, Herr, Van Vianen, & Schmidt, 2017) and as a continuum by others (Paulhus, 1998). Grandiosity and vulnerability are considered as the two expressions of narcissism (Cain, Pincus, & Ansell, 2008): grandiose narcissism is associated with the predisposition to exploit others, a lack of empathy and one's feelings of entitlement and superiority, whether vulnerable narcissism is associated with a depleted self‐image, social withdrawal and suicidality (Miller, Gentile, Wilson, & Campbell, 2013). The current gold‐standard models of narcissism, the trifucated model (Miller et al., 2016) and the narcissism spectrum model (Krizan & Herlache, 2018) postulate that grandiosity and vulnerability are two largely independent factors that are tied together by a core of entitlement.

The main tool used to study the construct of narcissism is the Narcissistic Personality Inventory (NPI; Raskin & Hall, 1979), which represents grandiose narcissism (Krizan & Herlache, 2018). The NPI consists of forty dichotomous items composed of both a narcissistic and a non‐narcissistic statement. The authors of the questionnaire propose seven domains of narcissism: *authority* reflects one's need for authority and success (e.g., item 33 “I would prefer to be a leader”); *exhibitionism* represents one's need to be the center of attention in a social context (e.g., item 30 “I like to be the center of attention”); *superiority* measures one's belief of being better than other people (e.g., item 40 “I am an extraordinary person”); *entitlement* reflects one's desire to receive respect and wield power (e.g., items 14 “I insist upon getting the respect that is due me” and 27 “I have a strong will to power”); *exploitativeness* represents one's capacity to manipulate other people (e.g., item 13 “I find it easy to manipulate people”); *self‐sufficiency* measures one's autonomy and belief in oneself (e.g., items 22 “I rarely depend on anyone else to get things done” and 34 “I am going to be a great person”); *vanity* measures one's admiration of one's own physical appearance (e.g., item 19 “I like to look at my body”). However, this seven‐domain structure of the NPI is controversial; several studies report different structures of the questionnaire, such as a four‐factor model (Emmons, 1987) and a three‐factor model (Boldero, Bell, & Davies, 2015).

Despite inconsistent results in the exploration of dimensionality (Ackerman et al., 2011; Corry, Merritt, Mrug, & Pamp, 2008; Kubarych et al., 2004), narcissism is commonly understood as composed of domains that are interchangeable measures of the construct proposed. In the last decade, a new way of analyzing psychological constructs as complex systems has been proposed: the network approach (Borsboom, 2017). Such complex systems are uncovered in empirical studies with network models, that represent a given construct as emerging from mutual interactions of its components (Borsboom & Cramer, 2013).

The network approach has been used to analyze a number of mental disorders, such as depression (Mullarkey, Marchetti, & Beevers, 2018), posttraumatic stress disorder (Fried et al., 2018; Phillips et al., 2018). Psychological constructs such as personality (Costantini et al., 2015), empathy (Briganti, Kempenaers, Braun, Fried, & Linkowski, 2018) and self‐worth (Briganti, Fried, & Linkowski, 2019) have also been proposed as network structures. The Pathological Narcissism Inventory has been recently investigated through the lens of network analysis (Di Pierro, Costantini, Benzi, Madeddu, & Preti, 2019), which identified Contingent self‐esteem, Grandiose Fantasies and Entitlement Rage to be important traits of the constructs.

A network can be composed of items of a questionnaire such as the NPI. In the case of a network of self‐reported questions, several items tend to be redundant and represent the same aspect of a construct; this has been described in the network literature as a delicate challenge, since the meaning of a connection between two redundant elements changes and simply represent shared variance between the two corresponding questions that measure the same thing (Fried & Cramer, 2017).

This challenge applies to the NPI: for instance, items 19 (“I like to look at my body”) and 29 (“I like to look at myself in the mirror”) are two very similar measurements from *vanity.* This is the case for several other items in the questionnaire, including items from different domains, such as items 12 (“I like to have authority over other people”) and 27 (“I have a strong will to power”) that respectively belong to *authority* and *entitlement.* In a network structure, we would expect these items to be strongly associated.

It is plausible to consider narcissism as a network of components (in this case, items from a self‐reported questionnaire indicating an individual's perspective on narcissistic traits) that mutually influence each other instead of being passive consequences of the same construct. The network approach to narcissism is relevant because it might allow in clinical samples the identification of meaningful targets for intervention, even more so if considered that normal and pathological narcissism form a continuum.

The aim of this work is to explore for the first time NPI items and their relationship in a network of narcissism, therefore applying network analysis to the items of the questionnaire. Network analysis has been shown to offer substantial insight as a complementary tool to factor analysis, which is a more established technique in the field of personality assessment (Briganti et al., 2018): as mentioned, modeling a construct or mental disorder as a network can highlight connections between items or symptoms which can therefore be used for intervention (Blanken et al., 2019).

First, we want to explore the connectivity of the NPI network. Second, we want to explore the importance of each item in the questionnaire using strength centrality, which is the absolute sum of connections of a given node in the network (Boccaletti, Latora, Moreno, Chavez, & Hwang, 2006).

## 2 METHOD

### 2.1 Participants

The dataset used for this study is composed of 942 university students from the French‐speaking region of Belgium. The participants were first‐year students in several Belgian universities and in different undergraduate courses and they volunteered to fill a set of questionnaires which included a French version of the NPI among other questionnaires such as the Interpersonal Reactivity Index (Briganti et al., 2018), the Contingencies of Self‐Worth Scale (Briganti et al., 2019), the Resilience Scale for Adults (Briganti & Linkowski, 2019), the Zung Depression Scale and the Toronto Alexithymia Scale. The questionnaire of the French version of the NPI is fully detailed in a previous paper (Braun, Kempenaers, Linkowski, & Loas, 2016).

### 2.2 Measurement

The NPI (Table 1) contains 40 items that are meant to assess seven domains of narcissism: *authority*, *exhibitionism*, *superiority*, *entitlement*, *exploitativeness*, *self‐sufficiency* and *vanity.* Items from different domains are shuffled in the questionnaire, and their scoring is dichotomous: each item possesses both a narcissistic and a non‐narcissistic statement. Our dataset is anonymized and is provided with the full R‐code in the supplementary materials to ensure total reproducibility of the analyses carried out in the paper. The protocol of this study was approved by the Ethical Committee of the Erasme university hospital.

**Table 1:** Narcissistic Personality Inventory

N°	0	1	Domain	Label
1	I am not good at influencing people	I have a natural talent for influencing people	Authority	A1
2	I am essentially a modest person	Modesty does not become me	Exhibitionism	Exh2
3	I tend to be a fairly cautious person	I would do almost anything on a dare	Exhibitionism	Exh3
4	When people compliment me I sometimes get embarrassed	I know that I am good because everybody keeps telling me so	Superiority	S4
5	The thought of ruling the world frightens the hell out of me	If I ruled the world it would be a better place	Entitlement	En5
6	I try to accept the consequences of my behavior	I can usually talk my way out of anything	Exploitativeness	Exp6
7	I prefer to blend in with the crowd	I like to be the center of attention	Exhibitionism	Exh7
8	I am not too concerned about success	I will be a success	Authority	A8
9	I am no better or worse than most people	I think I am a special person	Superiority	S9
10	I am not sure if I would make a good leader	I see myself as a good leader	Authority	A10
11	I wish I were more assertive	I am assertive	Authority	A11
12	I do not mind following orders	I like to have authority over other people	Authority	A12
13	I do not like it when I find myself manipulating other people	I find it easy to manipulate people	Exploitativeness	Exp13
14	I usually get the respect that I deserve	I insist upon getting the respect that is due me	Entitlement	En14
15	I do not particularly like to show off my body	I like to show off my body	Vanity	V15
16	People are sometimes hard to understand	I can read people like a book	Exploitativeness	Exp16
17	If I feel competent, I am willing to take responsibility for making decisions	I like to take responsibility for making decisions	Self‐sufficiency	SS17
18	I just want to be reasonably happy	I want to amount to something in the eyes of the world	Entitlement	En18
19	My body is nothing special	I like to look at my body	Vanity	V19
20	I try not to be a show off	I will usually show off if I get the chance	Exhibitionism	Exh20
21	Sometimes I am not sure of what I am doing	I always know what I am doing	Self‐sufficiency	SS21
22	I sometimes depend on people to get things done	I rarely depend on anyone else to get things done	Self‐sufficiency	SS22
23	Sometimes I tell good stories	Everybody likes to hear my stories	Exploitativeness	Exp23
24	I like to do things for other people	I expect a great deal from other people	Entitlement	En24
25	I take my satisfactions as they come	I will never be satisfied until I get all that I deserve	Entitlement	En25
26	Compliments embarrass me	I like to be complimented	Superiority	S26
27	Power for its own sake does not interest me	I have a strong will to power	Entitlement	En27
28	I do not care about new fads and fashions	I like to start new fads and fashion	Exhibitionism	Exh28
29	I am not particularly interested in looking at myself	I like to look myself in the mirror	Vanity	V29
30	It makes me uncomfortable to be the center of attention	I really like to be the center of attention	Exhibitionism	Exh30
31	People cannot always live their lives in term of what they want.	I can live my life in any way I want to	Self‐sufficiency	SS31
32	Being an authority does not mean that much to me	People always seem to recognize my authority	Authority	A32
33	It makes little difference to me whether I am a leader or not	I would prefer to be a leader	Authority	A33
34	I hope I am going to be successful	I am going to be a great person	Self‐sufficiency	SS34
35	People sometimes believe what I tell them	I can make anybody believe anything I want them to	Exploitativeness	Exp35
36	Leadership is a quality that takes a long time to develop	I am a born leader	Authority	A36
37	I do not like people to pry into my life for any reason	I wish somebody would someday write my biography	Superiority	S37
38	I do not mind blending into the crowd when I go out in public	I get upset when people do not notice how I look when I go out in public	Exhibitionism	Exh38
39	There is a lot that I can learn from other people	I am more capable than other people	Self‐sufficiency	SS39
40	I am much like everybody else	I am an extraordinary person	Superiority	S40

### 2.3 Network analysis

The software R (version 3.5.1, open source, available at https://www.r-project.org/) was used to carry out the analyses. We used the R‐packages “qgraph” (Epskamp, Cramer, Waldorp, Schmittmann, & Borsboom, 2012) and “glasso” (Friedman, Hastie, & Tibshirani, 2014) and IsingFit (van Borkulo et al., 2014) for network estimation and visualization, and “bootnet” (Epskamp, Borsboom, & Fried, 2017) for stability analyses. Complete information about package versions used in this paper is found in the supplementary materials.

### 2.4 Network estimation

An Ising Model (IM) was estimated from our dataset. An IM (Marsman et al., 2018; van Borkulo et al., 2014) is the binary equivalent of the Gaussian Graphical Model used for continuous datasets (Epskamp et al., 2017). A lasso (least absolute shrinkage and selection operator) was used to provide a conservative network structure (Epskamp & Fried, 2018). We used the default eLasso procedure which combines an l1‐regularized logistic regression with an Extended Bayesian Information Criterion (EBIC; Chen & Chen, 2008) which reports relevant connections between variables. The lasso procedure provides a neighborhood (set of nodes that interact) and decides the best set of regression coefficients given the data, based on EBIC (which is in turn based on log likelihood); the set of regression coefficients with the lowest EBIC is the best fit. To construct the final network, a connection is drawn between two nodes A and B if node A has node B in its set of neighbors and vice‐versa. The default eLasso procedure was used in bootnet and IsingFit (van Borkulo et al., 2014). The hyperparameter gamma (to select how many edges the model recovers) was set by default at 0.25; the optimal tuning parameter lambda (used to select the model with the best fit) was automatically chosen by the eLasso procedure. The network structure resulting from this estimation contains items from the NPI represented as nodes. An edge is a connection between two nodes in the network, which is interpreted as the existence of a connection between two nodes controlling for all other nodes in the network.

While estimating a network structure from items of a questionnaire, a connection between two nodes means that the observed group answers on average in a similar way to both items of the questionnaire (Briganti et al., 2018). Each edge in the network represents either a positive (visualized as blue edges) or a negative connection (visualized as red edges). The thickness and color saturation of an edge denotes its weight (the strength of the connection between two nodes).

The Fruchterman‐Reingold algorithm places the items in the network based on the inverse of the sum of connections of a given node with other nodes (Fruchterman & Reingold, 1991): this means that strongly connected nodes are put closer in the network visualization.

### 2.5 Network inference

We estimated strength centrality (Boccaletti et al., 2006) for the 40 items in the questionnaire. Strength centrality represents the absolute sum of the edges of a given node and therefore informs us of the connectedness of items in the network (Briganti et al., 2018).

### 2.6 Network stability

Stability analyses (Epskamp et al., 2017) were carried out through bootstrapping, which is a repeated estimation of a model under sampled data: we used 2000 bootstraps in this paper. An edge weight difference test was performed to compare all edges against all other edges and to answer the question “is edge A significantly stronger than edge B?”. Centrality stability analyses for strength centrality were also carried out to answer the question “is the centrality order stable?”. Centrality difference test was performed to answer the question “is the centrality estimate of node A statistically different from that of node B?”

We used the subsetting bootstrap procedure that re‐estimates the network with a dropping percentage of participants to determine the stability of centrality estimation, and results in a centrality‐stability coefficient (CS‐coefficient) that should not be lower than 0.25 and preferably above 0.5.

Both difference tests (edge weight and centrality) are carried out by estimating confidence intervals around the difference of two elements A and B (which are bootstrapped edge weights or bootstrapped centrality estimates, depending on the test): if 0 belongs in the confidence interval then there is no difference between A and B. Stability analyses in network analysis are explained in more detail in Epskamp et al. (2017).

## 3 RESULTS

### 3.1 Participants

Participants were 17 to 25 years old (*M* = 20 years, *SD* = 1.7 years), 55% of them were female and 45% were male. 25.4% of students studied engineering, 20% medicine, 17.7% economics, 11.3% sciences, 4.7% psychology and 2% law.

The average NPI score of the participants of this study was 13 (out of 40), and the standard deviation was 6.4.

### 3.2 Network of narcissism

**Figure 1:**
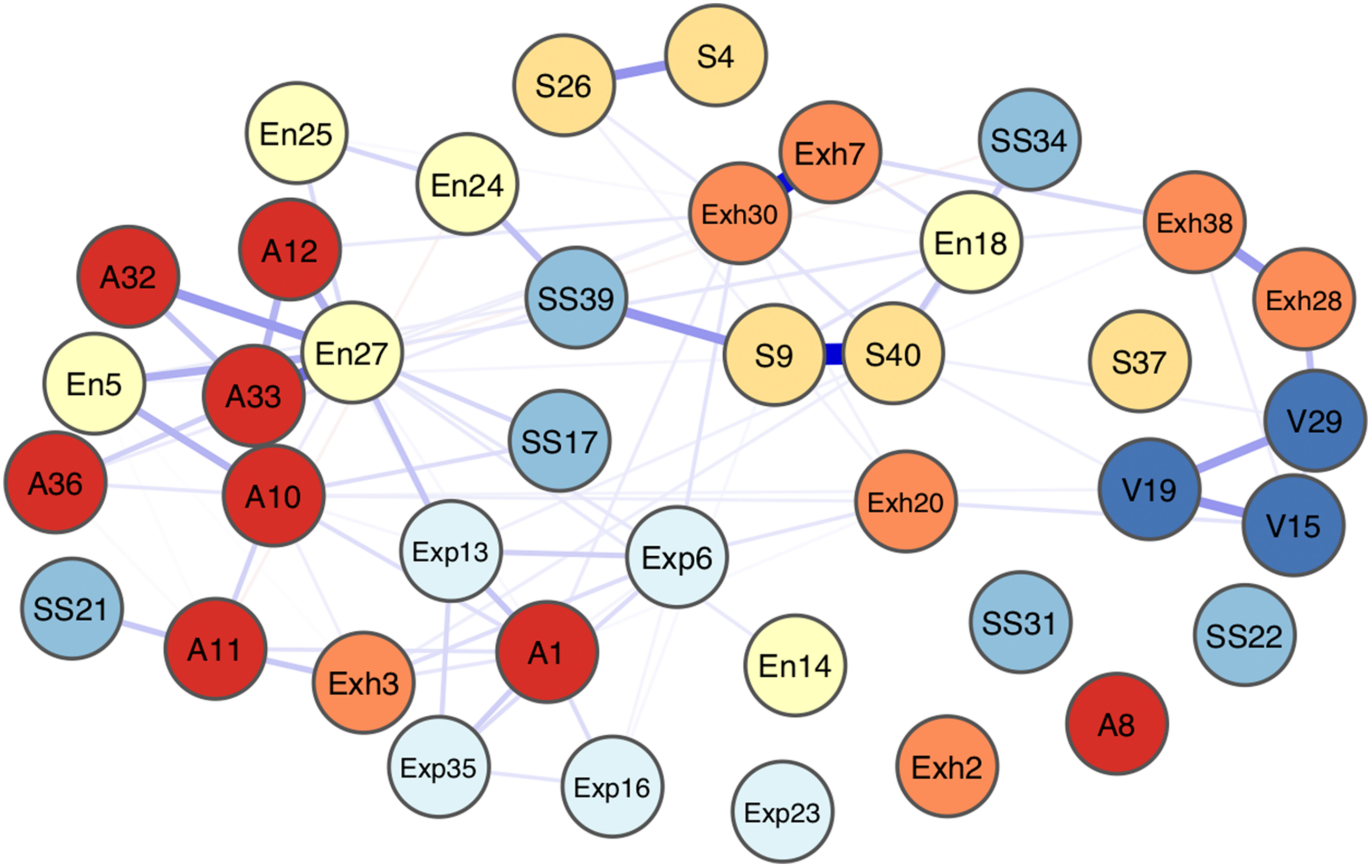
Network composed of the 40 items from the NPI (details in Table 1). Each item is represented by a node (1 to 40) and belongs to a different domain of the NPI (indicated by a color code). The name of each node is composed as following: an abbreviation of the domain to which the item belongs to followed by the item number

Figure 1 illustrates the estimated network of the 40‐item NPI. Overall, items from the NPI form a positively connected network. The strongest connections in the network are found between nodes belonging to the same domain of narcissism: for instance, item 10 (“I see myself as a good leader”) is strongly associated to item 33 (“I would prefer to be a leader”) and both belong to the *authority* domain; item 7 (“I like to be the center of attention”) presents the second strongest connection in the network to item 30 (“I really like to be the center of attention”) and both belong to *exhibitionism*; item 9 (“I think I am a special person”) shares the strongest edge of the network with item 40 (“I think I am an extraordinary person”) and both belong to the *superiority* cluster. In the case of these three connections, the items involved in an edge measure the same aspect of the construct.

Several connections are found between items belonging to different domains, and we want to illustrate some of these connections. Domains *superiority* and *self‐sufficiency* are connected through items 9 (“I think I am a special person”) and 39 (“I am more capable than other people”); domains *authority* and *entitlement* connect through items 12 (“I like to have authority over other people”) and 27 (“I have a strong will to power”); domains *authority* and *exploitativeness* connect through items 1 (“I have a natural talent for influencing people”) and 35 (“I can make anybody believe anything I want them to”). These domains also tend to measure the same thing, even though belonging to different domains. Some small, negative edge are also found in the network, such as the one between items 11 (“I am assertive”) and 24 (“I expect a great deal from other people”).

### 3.3 Network inference

**Figure 2:**
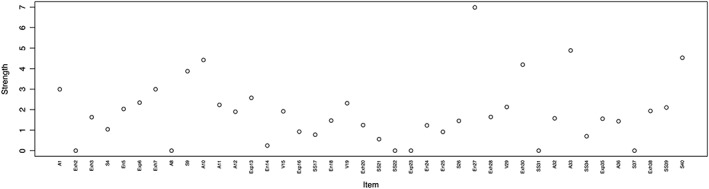
Strength centrality estimates for the 40 items of the NPI. The *Y*‐axis represents centrality indices (the higher the estimate the more central the item), and the *X*‐axis represents the 40 NPI items

Figure 2 shows strength centrality estimates for the 40‐item NPI. Item 27 from *entitlement* (“I have a strong will to power”) presents the highest strength estimate, which means that it is the most interconnected node in the network. Other strong items include item 33 from *authority* (“I would prefer to be a leader”) and item 40 from *superiority* (“I am an extraordinary person”). Several items present with a strength centrality of 0, which means that they are not connected with any item in the network.

### 3.4 Network stability

The edge weight bootstrap shows relatively narrow CIs, which indicates a precise estimation of the edge weights in the network. The edge‐weight difference test performed shows that stronger edges are significantly stronger than other edges in the network; however, edges 9–40 and 7–30 are not statistically different from each other, which means that, even though edge 9–40 reports a stronger connection in the network, we cannot safely interpret it to be statistically stronger than edge 7–30.

Strength centrality stability analyses report that the centrality order is relatively stable, with a centrality stability coefficient (CS‐coefficient) of 0.67 (for more information, see Briganti et al., 2018). Strength centrality difference test reports that stronger centrality estimates are significantly stronger than other estimates but are not significantly different from each other; for instance, we cannot infer whether the centrality of item 27 is really stronger than that of item 33. We obtained a CS‐coefficient of 0.67, which indicates stable results.

## 4 DISCUSSION

This study is to our knowledge the first application of network analysis to the NPI. Connections are shown between narcissistic domains and shed light on how they interact. Items from the NPI are overall positively connected and some items are more connected than others. Most items from the NPI share some variance and are connected. However, some items present weak connections with others; this means that some nodes are conditionally independent of all other items in the network. Connections exist both between items from the same domain and between items from domain, and stability analyses show that we can safely interpret connections in this study.

Several strong connections between items from the NPI are found in the network. In the case of the three connections between items 10–33, 7–30 and 9–40 belonging to the same domains (respectively *authority*, *exhibitionism* and *superiority*) as described in the Results section, the interpretation of an edge changes (Fried & Cramer, 2017), and the resulting connection simply represents shared variance between the two questions (since they measure the same thing). In some cases, items from different domains also tend to represent the same construct, such as items 12 and 27 that connect *authority* and *entitlement* in the network. These items can be considered as “bridge items”, since they can transfer information from one domain to another and vice‐versa; however, bridge items as the examples described in the *Results* section also tend to represent the same aspect of narcissism.

Centrality analysis shows that items from *entitlement*, *authority*, and *superiority* present the highest strength centrality estimates: that means that items from these domains connect well to a greater number of nodes in the network, therefore identifying these 3 domains as containing specific items that are important in this NPI network. From a network point of view, it is also not surprising to find *entitlement* to contain central items, as this finding supports our current gold standard models of narcissism, the trifucated model (Miller et al., 2016) and the narcissism spectrum model (Krizan & Herlache, 2018) that describe *entitlement* as a connection between grandiosity and vulnerability. Our finding also supports the recent network study of pathological narcissism (Di Pierro et al., 2019), which reported high centrality values for Entitlement Rage. In the network approach, if the observed group scores high on a highly central node, then the observed group is also more likely to score high on a relevant number of nodes in the network. The identification of central items may help in identifying potential targets for clinical intervention in people suffering from narcissism.

Our findings should be interpreted in the light of several limitations. First, our dataset is composed of university students, which limits the potential generalization of our findings to different samples. Second, because this is a cross‐sectional study, we cannot infer whether a given node (item or domain) causes or is caused by another node to which it is connected. Third, redundancy among items that measure the same thing is an important issue that has yet to be solved in psychological networks of self‐reported questionnaires; in the case of the NPI network, several items can be considered as redundant, which would alter the connectivity with other items (such as reported with strength centrality values).

Further studies may endeavor to replicate our findings in different samples, both non‐clinical and clinical, to identify central features of narcissism.

## CONFLICT OF INTERESTS

None.

## Supporting information

Data S1. Supporting information

Data S2. Supporting information

Data S3. Supporting information
