# Supplementary material for: Exploring network structure and central items of the Narcissistic Personality Inventory
Source: Int J Methods Psychiatr Res. 2019 Dec 5;29(1):e1810. doi: 10.1002/mpr.1810 (PMC7051847; doi:10.1002/mpr.1810)
Supplement: Supplementary file 2 — Data S2. Supporting information [file MPR-29-e1810-s002.docx]

**Details of the R Package used for the analysis**

"> sessionInfo()
R version 3.5.1 (2018-07-02)
Platform: x86_64-apple-darwin15.6.0 (64-bit) Running under: macOS 10.14.4

Matrix products: default
BLAS: /System/Library/Frameworks/Accelerate.framework/Versions/A/Frameworks/vecLib.framework/Versions/A/libBLAS.dylib
LAPACK: /Library/Frameworks/R.framework/Versions/3.5/Resources/lib/libRlapack.dylib

locale:
[1] en_US.UTF-8/en_US.UTF-8/en_US.UTF-8/C/en_US.UTF-8/en_US.UTF-8

attached base packages:
[1] grid parallel stats graphics grDevices utils datasets methods
[9] base

other attached packages:
[1] bindrcpp_0.2.2 Matrix_1.2-15 Hmisc_4.1-1 Formula_1.2-3
[5] survival_2.43-3 lattice_0.20-38 psych_1.8.12 data.table_1.12.0
[9] reshape2_1.4.3 bnlearn_4.4 RBGL_1.56.0 Rgraphviz_2.24.0
[13] graph_1.58.0 BiocGenerics_0.26.0 corpcor_1.6.9 ggm_2.3
[17] pcalg_2.6-0 dplyr_0.7.8 lavaan_0.6-3 glasso_1.10
[21] igraph_1.2.2 mgm_1.2-5 bootnet_1.1.0 ggplot2_3.1.0
[25] readr_1.3.1 qgraph_1.5

loaded via a namespace (and not attached):
[1] minqa_1.2.4 colorspace_1.4-0 rjson_0.2.20
[4] parcor_0.2-6 htmlTable_1.13.1 base64enc_0.1-3
[7] clue_0.3-56 rstudioapi_0.9.0 IsingFit_0.3.1
[10] longitudinal_1.1.12 mvtnorm_1.0-8 codetools_0.2-16
[13] splines_3.5.1 mnormt_1.5-5 robustbase_0.93-3
[16] knitr_1.21 jsonlite_1.6 nloptr_1.2.1
[19] cluster_2.0.7-1 sfsmisc_1.1-3 png_0.1-7
[22] compiler_3.5.1 backports_1.1.3 assertthat_0.2.0
[25] lazyeval_0.2.1 survey_3.35 acepack_1.4.1
[28] htmltools_0.3.6 tools_3.5.1 coda_0.19-2
[31] gtable_0.2.0 glue_1.3.0 V8_1.5
[34] Rcpp_1.0.0 statnet.common_4.2.0 relaimpo_2.2-3
[37] nlme_3.1-137 iterators_1.0.10 xfun_0.4
[40] stringr_1.3.1 network_1.13.0.1 lme4_1.1-19
[43] gtools_3.8.1 DEoptimR_1.0-8 MASS_7.3-51.1
[46] zoo_1.8-4 scales_1.0.0 BDgraph_2.53
[49] dagitty_0.2-2 hms_0.4.2 GeneNet_1.2.13
[52] NetworkToolbox_1.2.2 huge_1.2.7 RColorBrewer_1.1-2
[55] curl_3.3 pbapply_1.3-4 gridExtra_2.3
[58] IsingSampler_0.2 bdsmatrix_1.3-3 rpart_4.1-13
[61] fastICA_1.2-1 latticeExtra_0.6-28 Epi_2.32
[64] stringi_1.2.4 foreach_1.4.4 sem_3.1-9
[67] checkmate_1.9.1 boot_1.3-20 rlang_0.3.1
[70] pkgconfig_2.0.2 d3Network_0.5.2.1 arm_1.10-1
[73] purrr_0.2.5 bindr_0.1.1 labeling_0.3
[76] htmlwidgets_1.3 cmprsk_2.2-7 tidyselect_0.2.5
[79] plyr_1.8.4 magrittr_1.5 R6_2.3.0
[82] sna_2.4 pillar_1.3.1 whisker_0.3-2
[85] foreign_0.8-71 withr_2.1.2 mgcv_1.8-26
[88] ppls_1.6-1.1 abind_1.4-5 nnet_7.3-12
[91] tibble_2.0.1 etm_1.0.4 crayon_1.3.4
[94] fdrtool_1.2.15 ellipse_0.4.1 jpeg_0.1-8
[97] pbivnorm_0.6.0 matrixcalc_1.0-3 digest_0.6.18
[100] mi_1.0 tidyr_0.8.2 numDeriv_2016.8-1
[103] stats4_3.5.1 munsell_0.5.0 glmnet_2.0-16
[106] mitools_2.3"
